# 

**Published:** 1900-11-03

**Authors:** 


### NOTICE TO CORRESPONDENTS.

All MSS., Letters, Books for Review, and other matters intended for the Editor should be addressed The Editor, "The Hospital," The Hospital Building, 28 & 29 Southampton Street, Strand, W.C.
The Editor cannot undertake to return rejected MS., even when accompanied by stamped directed envelope.

**Notice to Advertisers and Subscribers.**
All Advertisements, Orders for Copies of The Hospital, and Business Communications should be addressed to **THE MANAGER (Not to the Editor),** The Hospital, The Hospital Building, 28 & 29 Southampton Street, Strand, London, W.C.
The Cost of Subscription, post free, is as follows:—Per week, 2½d.; per per quarter, 2s. 8d.; per half-year, 5s. 3d.; per annum, 10s. 6d. Foreign.—Per annum, 15s. 2d., payable, in all cases, in advance.

**NOTICE.—Vol. XXVIII. of "The Hospital" (April to September, 1900), in handsome cloth binding, will shortly be on sale at the Office of this Journal, price, 7s. 6d. Cases for binding the Numbers contained in this Volume 1s. 6d. Index to Vol. XXVIII., 2½d., post free.**
**The Monthly Part for November will be ready on November 1st. Price 6d., by post 8d.**

### BOOKS RECEIVED.

The Knickerbocker Press.

"The Ophthalmic History of an English School" (A.D. 1856 to 1900). By Sydney Stephenson.

John Bale, Son, and Danielsson.

"Life in an Open-air Sanatorium." By Dr. Charles Reinhardt.

T. Fisher Unwin.

"Thomas Sydenham." By Joseph Frank Payne, M.D. Oxon.

Longmans, Green and Co.

"Malaria according to the New Researches." By Professor Angelo Celli. Translated by John Joseph Eyre, M.R.C.P., L.R.C.S. Irel., D.P.H. Camb., with an Introduction by Dr. Patrick Manson.

S. W. Partridge & Co.

"The Zenana," Vol. VII.

L. Bruck., Sydney.

"The Australasian Medical Directory and Handbook." Edited and compiled by Ludwig Bruck.

G. W. Bacon.

Bacon's "Unrivalled" Parliamentary Map of the British Isles.

### Scraps and Gleanings.

During the past quarter 408 in-patients have been admitted to the Norfolk and Norwich Hospital. The daily average of in-patients was 147⅗.

The Finance Committee of the Victoria Infirmary, Glasgow, have received a special donation of £105 from Messrs. Wm. Dixon, Limited, to meet the deficit on the ordinary income and expenditure account for the past year.

Thirty-seven cases of typhoid fever were admitted into the Exeter Sanatorium during one month, as well as eight scarlet-fever patients. In the same period nine typhoid, nine scarlet-fever, and four diphtheria patients were discharged cured.

A shilling subscription in aid of the Kingston Victoria Hospital has been initiated by the proprietors of the *Surrey Comet*. The first list announces the receipt of 891½ shillings, and it is hoped that with this good start the amount required, £200, will soon be raised.

The representatives of the three bodies representing the districts of Gravesend, Northfleet, and Strood have given their verdict in favour of the scheme for providing a joint hospital for infectious diseases. The structural expenses and also the establishment expenses are to be paid by the three authorities according to rateable value.

The handsome sum of £1,150 was realised from the three days' bazaar held in aid of the Lytham Cottage Hospital. At the opening of the bazaar it was announced that Mr. Clifton, the owner of the property on which the cottage hospital stood, desired, subject to certain conditions, to make a free gift of the land to the hospital. This gift represents a sum of about £3,000 or £4,000.

The Charity Commissioners' proposed new scheme for the management of St. Bartholomew's Hospital, Rochester, has been issued. The institution is now officially described as of "Rochester" instead of "Chatham," as formerly. The new scheme provides that any person or persons making a bequest of £1,000 or upwards, or making a gift or gifts to this amount to the hospital, may in consideration thereof appoint any person or persons to be additional trustees of the charity, to hold office for life.

### The Student of Medicine.

### APPOINTMENTS.

Wm. Ainslie, M.B., Ch.B.Aberd., appointed Medical Officer for the Kington District and the Workhouse of the Kington Union. Wm. M. Bergin, M.B.Lond., M.R.C.S., appointed House-Surgeon to the Royal Eye Hospital, Southwark. E. H. Birchall, L.R.C.P., L.R.C.S.Edin., appointed Assistant Medical Officer to the Walton Workhouse of the West Derby Union. W. J. S. Bythell, B.A., M.B., Ch.B., appointed Anæsthetist to the Manchester Children's Hospital, Pendlebury. H. Chesson, L.R.C.P.Lond., M.R.C.S.Eng., appointed Assistant Medical Superintendent to the Hospital for Insane, Toowoomba, Queensland. F. R. S. Cosens, M.R.C.S., L.R.C.P.Lond., appointed District Medical Officer to the Honiton Union. William E. Frost, M.S., Ch.B., appointed House-Surgeon to the Edinburgh Royal Maternity and Simpson Memorial Hospital. Dundas Grant, M.A., M.D., F.R.C.S.Eng., appointed Surgeon to the Royal Academy of Music. R. N. Highmoor, M.B., C.M.Edin., appointed Medical Officer for the Fransham District of the Mitford and Launditch Union. William H. Hill, M.B., C.M., appointed Clinical Assistant in Dr. Smart's Wards in the Edinburgh Royal Infirmary. Norman W. Kater, M.B., Ch.M.Syd., appointed Demonstrator of Anatomy at the University of Sydney. J. H. Marsh, M.R.C.S., L.R.C.P., appointed Medical Officer for the Marton District of Rugby Union. W. Francis Mellersh, L.D.S., R.C.S.Eng., appointed Honorary Dental Surgeon to the Royal Cambridge Asylum for Soldiers' Widows. W. P. Montgomery, M.A.Oxon, B.S., M.B.Lond., F.R.C.S.Eng., appointed Honorary Assistant Surgeon to the Manchester Royal Infirmary. J. H. Naismith, M.B.Glas., appointed Medical Officer of Health for the Tow Law Urban District. T. H. O'Shaughnessy, M.D.R.U.I., appointed Assistant Medical Officer to the Nottingham Union Infirmary. T. A. Price, M.B., appointed Assistant Medical Officer at the Hospital for Insane, Goodna, Queensland. Robert Riddle, M.B., C.M.Edin., appointed Medical Officer of Health for the Sandbach Urban District. Miss M. H. G. Russell, L.R.C.P., L.R.C.S.Edin., L.F.P.S.Glasg., appointed Assistant Medical Officer to the Camberwell Infirmary. S. S. Simmons, M.R.C.S.Eng., L.R.C.P.Lond., appointed Medical Officer for the Ninth District of the Croydon Union. Frederick B. Simpson, M.B., Ch.B., appointed House-Surgeon to the Edinburgh Royal Maternity and Simpson Memorial Hospital. J. Thomas, M.R.C.S., L.R.C.P.Lond., appointed Medical Officer of Health for the Newcastle Emlyn Urban District. Robert Stevenson Thompson, M.D., B.Sc., appointed to the Chair of Practice of Medicine in Anderson's College Medical School, Glasgow. A. Murray Wood, M.B., Ch.B., appointed a Clinical Assistant in Professor Greenfield's Wards in the Edinburgh Royal Infirmary.

